# Regulation of Microtubule Nucleation in Mouse Bone Marrow-Derived Mast Cells by Protein Tyrosine Phosphatase SHP-1

**DOI:** 10.3390/cells8040345

**Published:** 2019-04-11

**Authors:** Anastasiya Klebanovych, Vladimíra Sládková, Tetyana Sulimenko, Věra Vosecká, Zuzana Rubíková, Martin Čapek, Eduarda Dráberová, Pavel Dráber, Vadym Sulimenko

**Affiliations:** 1 Department of Biology of Cytoskeleton, Institute of Molecular Genetics, Czech Academy of Sciences, CZ-142 20 Prague, Czech Republic; Anastasiya.Klebanovych@img.cas.cz (A.K.); vladimira.sladkova@img.cas.cz (V.S.); tetyana.sulimenko@img.cas.cz (T.S.); vera.vosecka@img.cas.cz (V.V.); zuzana.rubikova@img.cas.cz (Z.R.); Eduarda.Draberova@img.cas.cz (E.D.); 2 Light Microscopy Core Facility, Institute of Molecular Genetics, Czech Academy of Sciences, CZ-142 20 Prague, Czech Republic; martin.capek@img.cas.cz

**Keywords:** bone marrow-derived mast cells, cell activation, γ-tubulin complexes, microtubule nucleation, SHP-1 tyrosine phosphatase

## Abstract

The antigen-mediated activation of mast cells initiates signaling events leading to their degranulation, to the release of inflammatory mediators, and to the synthesis of cytokines and chemokines. Although rapid and transient microtubule reorganization during activation has been described, the molecular mechanisms that control their rearrangement are largely unknown. Microtubule nucleation is mediated by γ-tubulin complexes. In this study, we report on the regulation of microtubule nucleation in bone marrow-derived mast cells (BMMCs) by Src homology 2 (SH2) domain-containing protein tyrosine phosphatase 1 (SHP-1; *Ptpn6*). Reciprocal immunoprecipitation experiments and pull-down assays revealed that SHP-1 is present in complexes containing γ-tubulin complex proteins and protein tyrosine kinase Syk. Microtubule regrowth experiments in cells with deleted SHP-1 showed a stimulation of microtubule nucleation, and phenotypic rescue experiments confirmed that SHP-1 represents a negative regulator of microtubule nucleation in BMMCs. Moreover, the inhibition of the SHP-1 activity by inhibitors TPI-1 and NSC87877 also augmented microtubule nucleation. The regulation was due to changes in γ-tubulin accumulation. Further experiments with antigen-activated cells showed that the deletion of SHP-1 stimulated the generation of microtubule protrusions, the activity of Syk kinase, and degranulation. Our data suggest a novel mechanism for the suppression of microtubule formation in the later stages of mast cell activation.

## 1. Introduction

Mast cells play a crucial role in allergies, as well as in innate and adaptive immune responses. They express plasma membrane-associated high-affinity IgE receptors (FcεRIs), the aggregation of which by a multivalent antigen (Ag)-IgE complex triggers mast cell activation, resulting in their degranulation; in the release of inflammatory mediators, proteases, and lipid mediators; and in the production of various cytokines [1]. FcεRI crosslinking initiates a tyrosine phosphorylation of the FcεRI β- and γ-subunits by the Src family non-receptor kinase Lyn. This is followed by an enhanced activity of tyrosine kinase Syk from the Syk/Zap family and the phosphorylation of transmembrane adaptors, which organize and coordinate further signals, resulting in a Ca^2+^ efflux from the endoplasmic reticulum. A depletion of Ca^2+^ from the ER lumen induces a Ca^2+^ influx across the plasma membrane, leading to an enhancement of the free cytoplasmic Ca^2+^ concentration, a step which is substantial in further signaling events [2]. The tyrosine phosphorylation of numerous substrates is only transient and returns to baseline levels several minutes after receptor triggering. An important role in this process is played by protein tyrosine phosphatases [3].

Microtubules, built up from αβ-tubulin dimers, are important for mast cell degranulation since the movement of secretory granules depends on intact microtubules [4,5]. It has been reported that the activation of mast cells induces an increased formation of microtubules [5,6] and the transient generation of protrusions containing microtubules (microtubule protrusions) [7,8]. Moreover, the influx of Ca^2+^ plays a decisive role in microtubule remodeling [7,9]. The importance of motor proteins for the anterograde [8] and retrograde [10] transport of the granules along the microtubules of mast cells was also demonstrated. Although these data point to the necessity of the microtubule network for mast cell degranulation, the molecular mechanisms responsible for microtubule reorganization in activated mast cells are still largely unknown.

In mast cells, microtubules are dominantly nucleated from the centrosomes. One of the key components for microtubule nucleation is γ-tubulin, a highly conserved, albeit minor, member of the tubulin superfamily [11]. Together with other proteins named Gamma-tubulin Complex Proteins (GCPs), it assembles into γ-Tubulin Small Complex (γTuSC) and the γ-Tubulin Ring Complex (γ TuRC). γTuSC is composed of two molecules of γ-tubulin and one copy each of GCP2 and GCP3. In mammalian cells, γTuRCs comprise γTuSCs and additional GCPs, GCP4–6 [12,13].

Protein tyrosine kinases phosphorylate γ-tubulin or associated proteins and, in this way, could modulate γ-tubulin functions [6,14,15]. It has been reported that Src signaling, leading to the activation of the MEF/ERK pathway, regulates microtubule nucleation by the accumulation of γ-tubulin at the centrosome [16,17]. Recently, we have reported that tyrosine-phosphorylated proteins GIT1 and βPIX regulate microtubule nucleation in mast cells [18]. The identification of protein tyrosine phosphatases that regulate microtubule nucleation should help in the elucidation of the mechanisms involved in the transient microtubule formation during mast cell activation.

In this study, we examined the hypothesis that phosphotyrosine (P-Tyr) proteins associated with γ-tubulin could modulate microtubule nucleation in activated mast cells. We identified protein tyrosine phosphatase SHP-1, forming complexes with γ-tubulin complex proteins, as a negative regulator of microtubule nucleation from the centrosomes of bone marrow-derived mast cells (BMMCs). The regulation is due to changes in γ-tubulin accumulation. During an Ag-induced activation, SHP-1 modulates the activity of the Syk kinase and affects the organization of microtubules. Our data suggest a novel mechanism for the attenuation microtubule formation in the later stages of mast cell activation. In this way, Ag-induced signaling pathways leading to the degranulation could be regulated.

## 2. Materials and Methods

### 2.1. Reagents

Nocodazole, puromycin, dinitrophenyl-albumin (DNP-albumin), fibronectin, and 4-nitrophenyl N-acetyl-β-D-glucosaminide (4-NAG) were from Sigma-Aldrich (St. Louis, MO, USA). Hygromycin B and Fura-2-acetoxymethyl ester (Fura-2-AM) were purchased from Invitrogen (Carlsbad, CA, USA). Protein A Sepharose CL-4B and Glutathione Sepharose 4 Fast Flow were from GE Healthcare Life Sciences (Chicago, IL, USA). Protein G Plus Agarose and the SuperSignal WestPico Chemiluminescent reagents were from Pierce (Rockford, IL, USA). The protease-inhibitor mixture tablets (Complete EDTA-free) were from Roche Molecular Biochemicals (Mannheim, Germany), and polyethylenimine was from (Polysciences, Inc., Warrington, PA, USA). The SHP-1 inhibitors TPI-1 and NSC87877 were purchased from Axon Medchem BV (Groningen, The Netherlands) and Selleckchem (Munich, Germany), respectively. The inhibitor stocks (10 mM) were prepared in DMSO. The restriction enzymes were from New England Biolabs (Ipswich, MA, USA). The oligonucleotides were synthesized by Sigma-Aldrich.

### 2.2. Antibodies

The catalog numbers for the primary commercial antibodies (Abs) are shown in parentheses. Mouse monoclonal Abs (mAb) TU-30 (IgG1) and TU-31 (IgG2b) to γ-tubulin sequence 434–449 were described previously [19,20]. Rabbit Ab (T3195) to γ-tubulin sequence 433–451 and rabbit Ab (T5192) and mouse mAb GTU-88 (IgG1; T6557) to γ-tubulin sequence 38–53 were from the Sigma-Aldrich. Rabbit Abs to SHP-1 (sc-287), SHP-2 (sc-280), and GIT1 (sc-13961) and mouse mAbs to GCP4 (IgG1, sc-271876), GCP5 (IgG2b, sc-365837), and GCP6 (IgG1, sc-374063) were purchased from Santa Cruz Biotechnology (Dallas, TX, USA). Mouse mAbs SPE-7 (IgE, D8406) specific for DNP; PY-20 (IgG2b, P4110) to P-Tyr; and rabbit Abs to actin (A2066), GFP (G1544), and βPIX (HPA004744) were from Sigma-Aldrich. Mouse mAb 4G10 (IgG2b) to P-Tyr was from Upstate Laboratories (Syracuse, NY, USA). Rabbit Abs to phospho-SHP-1 (Y^564^) (8849) and phospho-Syk (Y^352^) (2717) were from Cell Signaling (Danvers, MA, USA), to GCP3 (15719–1-AP) was from ProteinTech (Manchester, UK), and to α-tubulin (GTX15246) was from Genetex (Irvine, CA, USA). Mouse mAb to pericentrin (IgG1, 611815) was purchased from BD Transduction Laboratories (San Jose, CA, USA). Mouse mAbs GCP2–01 (IgG2b) and GCP2–02 (IgG1) to GCP2 were described previously [21]. Rabbit Ab to non-muscle myosin heavy chain (BT-561; Biomedical Technologies., Stoughton, MA, USA) and mAb NF-09 (IgG2a) to neurofilament NF-M protein [22] served as negative controls in the immunoprecipitation experiments. Mouse mAb Syk-01 (IgG1) [23] and rabbit Ab to Syk [24] were provided by Dr. Petr Dráber (Institute of Molecular Genetics CAS, Prague, Czech Republic).

Anti-mouse and anti-rabbit Abs conjugated with horseradish peroxidase (HRP) were from Promega Biotec (Madison, WI, USA). TrueBlot anti-rabbit IgG HRP was purchased from Rockland Immunochemicals (Limerick, PA, USA). Anti-mouse Ab conjugated with DyLight549 and anti-rabbit Ab conjugated with Cy3 or AF488 were from Jackson Immunoresearch Laboratories (West Grove, PA, USA).

### 2.3. Cell Cultures and Transfection

A stable cell line of mouse bone marrow-derived mast cells (BMMCs) was donated by Dr. Margaret Hibbs (Ludwig Institute for Cancer Research, Melbourne, Australia) [25]. The cells were cultured in freshly a prepared culture medium (RPMI-1640 supplemented with 100 U/mL penicillin, 100 µg/mL streptomycin, nonessential amino acids, 1 mM sodium pyruvate, 10% fetal calf serum, and 10% WEHI-3 cell supernatant as a source of IL-3). The cells were grown at 37 °C in 5% CO_2_ in air and passaged every 2–3 days.

The human epithelial breast cancer cells MCF7 (Cat. No. ATTC HTB-22) were obtained from the American Type Culture Collection (Manassas, VA, USA), and the human embryonic kidney HEK 293FT cells (HEK; Cat. No. R70007) were from ThermoFisher Scientific (Waltham, MA, USA). The cells were grown at 37 °C in 5% CO_2_ in Dulbecco’s Modified Eagle Medium supplemented with 10% fetal calf serum and antibiotics. The HEK cells used for lentivirus production were at passages 4–15. In some cases, the cells were cultivated with 100 nM TPI-1, 500 nM NSC87877, or a DMSO carrier (Control) for 1 h to evaluate the effect of SHP-1 inhibitors.

The HEK cells were transfected with 17 μg of DNA per 9-cm tissue culture dish using 51 μg of polyethylenimine and serum-free DMEM. After 24 h, the transfection mixture was replaced with a fresh medium supplemented with serum, and the cells were incubated for an additional 24 h.

### 2.4. DNA Constructs

To prepare C-terminally enhanced green fluorescent protein (EGFP)-tagged mouse SHP-1 (gene *Ptpn6*; RefSeq ID: NM_013545.3), the coding sequence without a stop codon was amplified from the C-terminally Myc-DDK-tagged Ptpn6 (tv1) (OriGene Technologies, Rockville, MD, USA; MR209258) by PCR using the forward 5′-GCTCGAATTCATGGTGAGGTGGTTTC-3′ and reverse 5′-AGCGTCGACCTTCCTCTTGAGAGAACCT-3′ primers. The PCR product was digested with *Eco*RI*/Sal*I and ligated to pEGFP-N3 (ClonTech Laboratories, Mountain View, CA, USA), resulting in plasmid pmSHP-1_EGFP.

To prepare a lentiviral vector for the phenotypic rescue experiment, the coding sequence of mouse SHP-1 was amplified from the Myc-DDK-tagged Ptpn6 (tv1) (OriGene Technologies, MR209258) by PCR using the forward 5′-AGAGCTAGCATGGTGAGGTGGTTTCACCGG-3′ and reverse 5′-AATGCGGCCGCTTACTTCCTCTTGAGAGAAC-3′ primers. The PCR product was digested with *Nhe*I*/Not*I and ligated into the pCDH-CMV-MCS-EF1-hygro vector (System Biosciences, Palo Alto, CA, USA), resulting in the lentiviral construct pmSHP-1-hygro.

To prepare N-terminally glutathione S-transferase (GST)-tagged mouse spleen-associated tyrosine kinase (gene *Syk*; RefSeq ID: NM_011418), the coding sequence was amplified from the C-terminally Myc-DDK-tagged Syk (tv1) (OriGene Technologies, MR209591) by PCR using the forward 5′-TCACGAATTCATGGCGGGAAGTGCTGTGGACA-3′ and reverse 5′-GGCCGTCGACTTAGTTAACCACGTCGTAGTAG-3′ primers. The PCR product was digested with *Eco*RI*/Sal*I and ligated into pGEX-6P-1 (Amersham Biosciences, Freiburg, Germany), resulting in the plasmid pGST-mSyk.

CRISPR/Cas9 gene editing [26] was used to disrupt the expression of all mouse SHP-1 variants (Ensembl, Ptpn6 ENSMUSG00000004266). Plasmids SpCas9 and pU6-sgRNAnew-III (donated by Dr. R. Malík, Institute of Molecular Genetics CAS, Prague, Czech Republic) were used for an optimal production of Cas9 and single-guide RNA (sgRNA), respectively. The CRISPR tool (available from Dr. F. Zhang Laboratory, Broad Institute, Cambridge, MA, USA) was used to design the DNA oligonucleotides (for production of sgRNA) that were cloned into the *BsmBI* sites of pU6-sgRNAnew-III. To enrich cells with a disrupted expression of SHP-1, we used the pRR-puro plasmid with multiple cloning sites that encode a nonfunctional puromycin resistance cassette [27]. Annealed sense and antisense oligonucleotides containing the sequences from the region of interest and overhangs with *Aat*II*/Sac*I restriction sites were ligated into pRR-puro digested with *Aat*II/*Sac*I, resulting in a reporter plasmid pRR-mSHP-1-puro. A co-transfection of the reporter plasmid with the plasmids encoding sgRNAs and Cas9 led to a CRISPR-induced double-strand break (DSB) in the reporter plasmid. When the DSB was repaired by a homologous recombination, puromycin resistance was restored.

### 2.5. Generation of SHP-1 Deficient Cell Lines

In order to delete part of the 5’ region of the gene containing the canonical and alternative start codons, BMMCs were transfected with CRISPR/Cas9 vectors (sgRNA#1, sgRNA#2, SpCas9) together with the reporter plasmid pRR-mSHP-1-puro by nucleofection using a Mouse Macrophage Kit and program T-020 in Amaxa Nucleofector II (Lonza Cologne AG, Cologne, Germany) according to the manufacturer’s instructions. After nucleofection, the cells were transferred into the culture media supplemented with 10% WEHI-3 cell supernatant (source of IL-3). Puromycin (5 µg/mL) was added into the culture media 48 h after nucleofection. A stable selection was achieved by culturing cells for 1 week in the presence of puromycin. The single-cell dilution protocol [28] was used to obtain cell clones that were thereafter analyzed by PCR and immunoblotting.

Single-cell clones were expanded, the genomic DNA was extracted with the QIAamp DNA Mini Kit (Qiagen, Gilden, Germany), and a deletion in the gene was determined by PCR amplification with primers flanking the deleted region: forward 5′-CAGAGTCCCATTGGTTTGACAGGCT-3′; reverse 5’-GGACAGGGGATTGGTTAGATACA-3′. The amplified fragments were visualized in 2% agarose gels stained by GelRed Nucleic Acid Gel Stain (Biotium, Fremont, CA, USA). While an amplification of short fragments (approx. 560bp) was detected in SHP-1 deficient clones, no amplification was found in control BMMCs due to the large size of the deleted region (approx. 6 kb).

### 2.6. Reverse Transcription PCR (RT-PCR)

The total RNAs from BMMCs or mouse spleen, heart, brain, and liver was isolated with the RNeasy Mini kit (Qiagen) and converted to cDNAs using the SuperScript^®^ VILO cDNA Synthesis Kit with random primers (ThermoFisher Scientific), according to the manufacturer’s protocol. The PCRs were performed with gene-specific primers for mouse SHP-1 (*Ptpn6*, NCBI Ref. Seq.: NM_013545.3, NM_001077705.2; primers anneal to both transcript variants) and for mouse SHP-2 (*Ptpn11*, NCBI Ref. Seq.: NM_011202.3, NM_001109992.1; primers anneal to both transcript variants). All primers were tested in silico by Basic Local Alignment Search Tool from National Center for Biotechnology Information (BLAST NCBI; NIH, Bethesda, MD, USA) to amplify the specific targets. The primer sequences are summarized in Supplemental Table S1. The PCRs were performed as described [20]. The amplified fragments were visualized in 2% agarose gels.

### 2.7. Real-Time qRT-PCR

The total RNAs was extracted in three independent isolations from nonactivated and activated BMMCs or SHP-1_KO cells using the RNeasy Mini Kit (QIAGEN), according to the manufacturer’s protocol. The RNAs were converted to cDNA with the High-Capacity cDNA Reverse Transcription Kit using random primers (Applied Biosystems, Waltham, MA, USA), according to the manufacturer’s protocol. The quantitative PCRs were performed with gene-specific primers for mouse interleukin 13 (*Il13*, NCBI Ref. Seq.: NM_008355.3), tumor necrosis factor (*Tnf*, NCBI Ref. Seq.: NM_013693.3, NM_001278601.1; primers anneal to both transcript variants), prostaglandin-endoperoxide synthase 2 (*Ptgs2*, NCBI Ref. Seq.: NM_011198.4), and glyceraldehyde-3-phosphate dehydrogenase (*Gapdh*, NCBI Ref. Seq.: NM_001289726.1, NM_008084.3; primers anneal to both transcript variants). All primers were tested in silico by NCBI BLAST to amplify the specific targets. The primer sequences are summarized in Supplemental Table S1. The quantitative PCRs were performed in the LightCycler 480 System (Roche, Mannheim, Germany) as described previously [20]. Each sample was run in triplicate. The identity of the PCR products was verified by sequencing.

### 2.8. Lentiviral Infection

Lentiviral infections were done as described previously [7] using HEK 293FT packaging cells for virus preparation. Virus particles with the pmSHP-1-hygro construct were added to the cells and replaced after 3 days with a fresh complete medium containing 1 mg/mL hygromycin B. A stable selection was achieved by culturing the cells for 1–2 weeks.

### 2.9. Cell Activation

The cells were sensitized with DNP-specific IgE (mouse mAb SPE-7; 1 μg/mL) for 2 h in a culture medium without the 10% WEHI-3 cell supernatant and activated with Ag (DNP-albumin conjugate; 100 ng/mL; 30–40 mol DNP/mol albumin) for 1–30 min in a buffered saline solution (20 mM HEPES, pH 7.4, 135 mM NaCl, 5 mM KCl, 1.8 mM CaCl_2_, 2 mM MgCl_2_, 5.6 mM glucose) supplemented with 0.1% albumin as described [7]. For the immunofluorescence experiments, the BMMCs were sensitized in suspension, overlaid on fibronectin-coated coverslips, and then activated [7].

### 2.10. Degranulation Assay and Determination of Intracellular Ca^2+^ Concentration

The degree of degranulation was quantified as the release of β-hexosaminidase from Ag-activated cells, using 4-NAG as a substrate [5]. The extent of degranulation was calculated as follows: absorbance of culture supernatant × 100/absorbance of total cell lysate and normalized to control cells.

Changes in the level of free intracellular Ca^2+^ were measured using Fura-2-AM as a cell permeant calcium reporter following the protocol for sample handling as described [7]. The intracellular free Ca^2+^ was measured in a microplate reader Infinite M200 (Tecan, Männedorf, Switzerland) as a ratio of Fura emissions at 510 nm after excitation with 340 nm and 380 nm (340/380) lasers at the indicated time points. After the measurement of the Ca^2+^ basic level, activation was triggered by the addition of Ag.

### 2.11. Evaluation of Cell Growth

Cell proliferation was assessed by the manual cell counting of control BMMCs or SHP-1_KO cells. A total of 2 × 10^5^ cells diluted in the culture medium were plated into the wells of a 6-well plate. The cells were counted at one day intervals from one to six days. The samples were counted in doublets in a total of three independent experiments.

### 2.12. Preparation of Cell Extracts

Whole-cell extracts for SDS-PAGE were prepared by washing the cells in a cold HEPES buffer (50 mM HEPES pH 7.6, 75 mM NaCl, 1 mM MgCl_2_, and 1 mM EGTA), solubilizing them in a hot SDS-sample buffer and boiling for 5 min. When preparing the extracts for immunoprecipitation and GST pull-down assays, the cells were rinsed twice in cold HEPES buffer and extracted at a concentration of 1 × 10^7^ cells/mL for 10 min at 4 °C with a HEPES buffer supplemented with 1% NP-40 (extraction buffer) and protease inhibitor mixture. The suspension was then spun down (20,000 × g, 15 min, 4 °C), and the supernatant was collected. When preparing the extracts for gel filtration chromatography, the cells were extracted at a concentration of 14 × 10^7^ cells/mL.

### 2.13. Immunoprecipitation, Kinase Assay, GST Pull-Down Assay, Gel Electrophoresis, and Immunoblotting

Immunoprecipitation was performed as previously described [29,30]. The cell extracts were incubated with beads of protein A saturated with mAbs to (i) γ-tubulin (TU-31; IgG2b), (ii) GCP2 (GCP2-01; IgG2b), or (iii) NF-M (NF-09; IgG2a, negative control); with rabbit Abs to (iv) γ-tubulin (T5192), (v) SHP-1, (vi) SHP-2, (vii) Syk, (viii) (GFP), (ix) GIT1, (x) βPIX, or (xi) non-muscle myosin (negative control); or with (xii) immobilized protein A alone. The extracts were also incubated with beads of protein G saturated with mAb to GCP4 (IgG1) or with immobilized protein G alone. The antibodies to γ-tubulin (T5192), SHP-1, SHP-2, GCP4, and GFP were used at Ig concentrations 2–5 µg/mL. Abs to Syk, in the form of ascitic fluid, and myosin were used at a dilution of 1:500 and 1:100, respectively. mAbs TU-31, GCP2-01, and NF-09, in the form of hybridoma supernatants, were diluted 1:2.

Alternatively, beads with immunoprecipitated material were used for the in vitro kinase assay as described previously [6]. The ^32^P-labeled-immunocomplexes were separated by gel electrophoresis and blotted to membranes, and ^32^P-labeled proteins were detected by autoradiography using the Amersham Typhoon scanner (GE Healthcare Europe GmbH, Freiburg, Germany). The preparation and purification of GST-tagged fusion proteins were described previously, as were the pull-down assays with whole-cell extracts [30].

Gel electrophoresis and immunoblotting were performed using standard protocols [31]. For immunoblotting, mAbs to γ-tubulin (GTU-88), P-Tyr (PY-20), GCP4, GCP5, and GCP6 were diluted 1:10,000, 1:2000, 1:1000, 1:1000, and 1:500, respectively. mAbs to GCP2 (GCP2-02), in the form of a spent culture supernatant, and Syk (Syk-01), in the form of ascitic fluid, were diluted 1:10 and 1:1000, respectively. Rabbit Abs to SHP-1, SHP-2, actin, GFP, GCP3, βPIX, and GIT1 were diluted 1:100,000, 1:50,000, 1:10,000, 1:5000, 1:3000, 1:3000, and 1:1000, respectively. Rabbit Abs to phospho-SHP-1 (Y^564^) and phospho-Syk (Y^352^) were diluted 1:2000 and 1:1000, respectively. Secondary anti-mouse and anti-rabbit Abs conjugated with HRP (Promega Biotec) were diluted 1:10,000. The TrueBlot anti-rabbit IgG HRP was diluted 1:100,000. The HRP signal was detected with SuperSignal WestPico Chemiluminescent reagents and the LAS 3000 imaging system (Fujifilm, Düsseldorf, Germany). The AIDA image analyzer v5 software (Raytest, Straubenhardt, Germany) was used for the quantification of signals from immunoblots.

### 2.14. Gel Filtration Chromatography

Gel filtration was performed using fast protein liquid chromatography (AKTA-FPLC system, Amersham) on a Superose 6 10/300 GL column (Amersham) as described previously [32]. The column equilibration and chromatography were performed in an extraction buffer.

### 2.15. Microtubule Regrowth

Microtubule regrowth from the centrosomes was followed in a nocodazole washout experiment. The cells growing in suspension were treated with nocodazole at a final concentration of 10 μM for 1 h at 37 °C to depolymerize microtubules. The cells were then washed with phosphate-buffered saline (PBS) precooled to 4 °C (3 times, 5 min each) to remove the drug and transferred to the complete medium tempered to 28 °C, and microtubule regrowth was allowed for 1.5 min at 28 °C. After that, the cells were fixed and immunostained in suspension. In the case of MCF7 growing on coverslips, the microtubules were depolymerized by 30 µM nocodazole for 1 h and regrowth was performed as described [33].

### 2.16. Immunofluorescence Microscopy

The cells were fixed and immunostained as described [34]. The samples were fixed in formaldehyde/Triton X-100 (F/Tx) and, for the double-label experiments with anti-γ-tubulin Ab, were postfixed in cold methanol (F/Tx/M). mAbs to P-Tyr (4G10) and pericentrin were diluted 1:1000 and 1:250, respectively. mAbs to γ-tubulin (TU-30), in the form of a spent culture supernatant, and Syk (Syk-01), in the form of ascitic fluid, were diluted 1:10 and 1:1000, respectively. Rabbit Abs to γ-tubulin (T3195) and α-tubulin were diluted 1:500 and 1:100, respectively. Secondary AF488- and Cy3-conjugated anti-rabbit Abs were diluted 1:200 and 1:1000, respectively. The DY549-conjugated anti-mouse Ab was diluted 1:1000. The preparations were mounted in MOWIOL 4–88 (Calbiochem, San Diego, CA, USA) and examined with an Olympus AX-70 Provis microscope (Olympus, Hamburg Germany) equipped with a 60×/1.0 NA water objective or with a Delta Vision Core system (AppliedPrecision, Issaquah, WA, USA) equipped with a 60×/1.42 NA oil objective. Some images were deconvoluted with Huygens Professional v18.10 (Scientific Volume Imaging, Hilversum, the Netherlands). To quantify the microtubule regrowth, different areas per sample were taken in both fluorescence channels. The sum of the γ-tubulin or α-tubulin immunofluorescence intensities was obtained from 11 (BMMCs) or 9 (MCF7) consecutive frames (0.2 μm steps), with the middle frame chosen with respect to the highest γ-tubulin intensity. The quantification of the microtubule regrowth assay was analyzed automatically in 1-μm regions of interest (ROIs) centered at the centrosomes, marked by γ-tubulin staining, using an in-house written macro (Supplemental Text 1) for Fiji processing program [35]. The determination of the number of cells that responded to the activation events by a generation of microtubule protrusions was done as described previously. Three experiments were performed, and in each experiment, 150–200 cells were examined [7].

### 2.17. Statistical Analysis

All data are presented as means ± SD or SE, as indicated. For the statistical analysis, the two-tailed, unpaired Student’s *t*-test was applied. For the statistical analysis of cells with microtubule protrusions, the Chi-Square Goodness of Fit Test was applied.

## 3. Results

### 3.1. Protein Tyrosine Phosphatase SHP-1 Interacts with Proteins of γ-Tubulin Complexes

**Figure 1:**
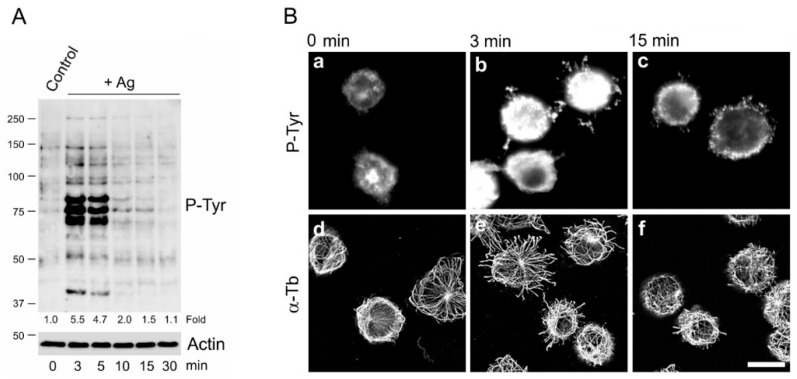
The antigen-induced activation of bone marrow-derived mast cells (BMMCs) results in a transient increase of the phosphorylation of proteins on tyrosine and the spatial redistribution of microtubules. (**A**) A comparison of the protein tyrosine phosphorylation level (P-Tyr) on blots from whole-cell lysates of controls and cells activated by FcεRI aggregation (+Ag) using an antigen (100 ng/mL) for various time intervals: Actin served as the loading control. Representative image, out of three repetitions, is shown. The numbers under the blot indicate the relative amounts of P-Tyr normalized to unstimulated control cells and the amount of actin in individual samples. (**B**) IgE-sensitized cells activated with antigen (100 ng/mL) for various time intervals were fixed and stained for tyrosine-phosphorylated proteins (**a**–**c**) and α-tubulin (**d**–**f**). Fixation F/Tx. Scale bar, 10 µm.

Ag-induced FcεRI aggregation results in a transient increase of the protein tyrosine phosphorylation level in mast cells isolated from different sources [36,37]. Similarly, in the BMMC line used in this study, the P-Tyr signal in Ag-activated cells substantially increased at the beginning of activation but then gradually weakened to the original level (Figure 1A). When compared with nonactivated cells, a 3-min activation resulted in an approximate fivefold increase of P-Tyr proteins, but after 30 min, the level of protein tyrosine phosphorylation returned to that in nonactivated cells (Supplemental Figure S1). Transient changes in the P-Tyr level after Ag-induced activation were also detected by immunofluorescence in fixed BMMCs stained with anti-P-Tyr antibody (Figure 1Ba–c). Previously, we have shown that an Ag-induced activation of BMMCs resulted in the generation of protrusions containing microtubules (microtubule protrusions) [7]. We observed a time course correlation between the level of P-Tyr signal and the generation of protrusions containing microtubules. In nonactivated cells, microtubule protrusions were very rare, but after 3 min activation, they appeared in a large number of cells. In later stages of the activation, microtubule protrusions diminished (Figure 1Bd–f). A statistical evaluation of BMMCs with microtubule protrusions during the activation was documented by a histogram (Supplemental Figure S3E, Control). Altogether, these data on BMMCs support the previous findings that active tyrosine phosphatases are involved in the later stages of an Ag-induced activation [3]. Protein tyrosine phosphatases might also be important for the regulation of microtubule organization in BMMCs.

**Figure 2:**
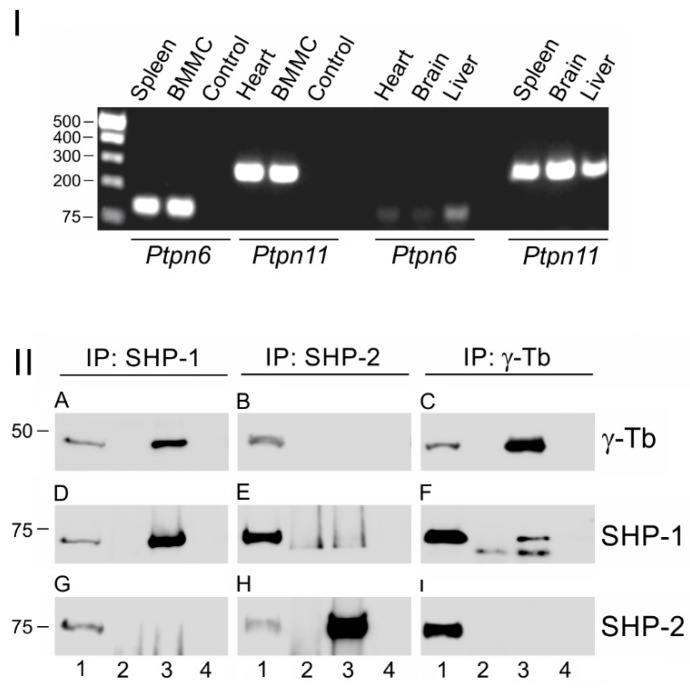
The SHP-1 and SHP-2 in complexes containing γ-tubulin. (**I**) The expression profile of SHP-1 and SHP-2 in selected mouse tissues and BMMCs: A gel-based RT-PCR analysis of mouse SHP-1 (*Ptpn6*) and SHP-2 (*Ptpn11*) is shown. Mouse spleen and heart served as positive controls for SHP-1 and SHP-2, respectively. (**II**) The extracts from BMMCs precipitated with immobilized Abs specific to SHP-1 (**A**,**D**,**G**), SHP-2 (**B**,**E**,**H**), or γ-tubulin sequence 434–449 (**C**,**F**,**I**): The blots were probed with Abs to γ-tubulin (γ-Tb), SHP-1, and SHP-2. The load (*lane 1*), the immobilized Abs not incubated with cell extracts (*lane 2*), the precipitated proteins (*lane 3*), and the carriers without Abs and incubated with cell extracts (*lane 4*).

It is well established that non-transmembrane Src homology 2 (SH2) domain-containing protein tyrosine phosphatases (SH-PTPs; SHPs) are critical regulators of intracellular signaling in activated mast cells [3]. SHP-1 (gene *Ptpn6*) and SHP-2 (gene *Ptpn11*) are SHPs sharing many structural and regulatory features. These include the presence of two SH2 domains (N-SH2, C-SH2), a phosphatase domain, and a C-terminal domain containing conserved tyrosine phosphorylation sites that influence the function and activities of these phosphatases [38]. The expression of SHPs in BMMCs was determined by gel-based RT-PCR analysis using mouse spleen and heart as positive controls for SHP-1 and SHP-2, respectively. The brain and the liver were used as additional positive controls for SHP-2. We found that both SHP-1 and SHP-2 were abundant in BMMCs. In the tested tissue samples, SHP-1 was prominent only in the spleen, while SHP-2 was highly expressed in all four tested mouse tissues: spleen, heart, brain, and liver (Figure 2I), confirming that SHP-2 is more general than SHP-1, which is primarily expressed in hematopoietic cells [38]. The immunoprecipitation experiments with Abs to SHP-1 and SHP-2 revealed a coprecipitation of γ-tubulin with SHP-1 (Figure 2IIA, lane 3) but not with SHP-2 (Figure 2IIB, lane 3). The reciprocal precipitation with mAb to γ-tubulin sequence 434–449 (TU-31) confirmed the interaction of SHP-1 (Figure 2IIF, lane 3) but not SHP-2 (Figure 2III, lane 3) with γ-tubulin. Similarly, when the Ab to γ-tubulin sequence 38–53 was used for the precipitation of the two SHP isotypes, only SHP-1 coprecipitated with γ-tubulin (Supplemental Figure S2A). In the following experiments, mAb TU-31 was used for the precipitation of γ-tubulin. The precipitation with Ab to SHP-1 did not coprecipitate SHP-2 (Figure 2IIG, lane 3) and with Ab to SHP2 did not coprecipitate SHP-1 (Figure 2IIE, lane 3). The results of these experiments suggested that SHP-1 might interact with γ-tubulin complexes and might modulate microtubule nucleation.

**Figure 3:**
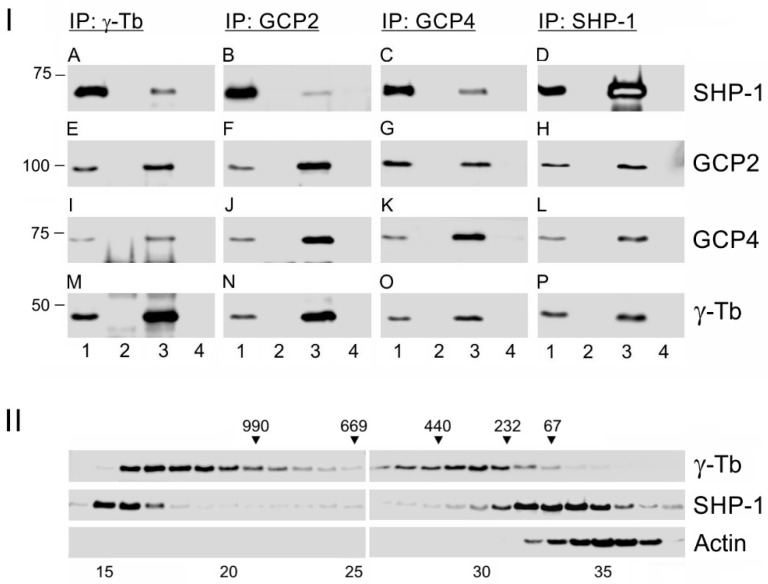
The SHP-1 associates with γ-tubulin complex proteins. (**I**) The immunoprecipitation experiments: Extracts from the BMMCs were precipitated with immobilized Abs specific to γ-tubulin (**A**,**E**,**I**,**M**), GCP2 (**B**,**F**,**J**,**N**), GCP4 (**C**,**G**,**K**,**O**), or SHP-1 (**D**,**H**,**L**,**P**). The blots were probed with Abs to SHP-1, GCP2, GCP4, and γ-tubulin (γ-Tb). The load (*lane 1*), the immobilized Abs not incubated with cell extracts (*lane 2*), the precipitated proteins (*lane 3*), and the carriers without Abs and incubated with cell extracts (*lane 4*). (**II**) The size distribution of the proteins fractionated on Superose 6: The blots of the collected fractions were probed with Abs to γ-tubulin (γ-Tb), SHP-1, and actin. The calibration standards (in kDa) are indicated on the top. The numbers at the bottom denote individual fractions.

To ascertain whether, besides γ-tubulin, SHP-1 interacts also with γ-tubulin complex proteins, immunoprecipitation experiments were performed with Abs to γ-tubulin, GCP2, and GCP4. The immunoblot analysis revealed a coprecipitation of SHP-1 with γ-tubulin (Figure 3IA, lane 3), GCP2 (Figure 3IB, lane 3), and GCP4 (Figure 3IC, lane 3). Also, the reciprocal precipitation with Ab to SHP-1 confirmed the interaction of GCP2 (Figure 3IH, lane 3), GCP4 (Figure 3IL, lane 3), and γ-tubulin (Figure 3IP, lane 3) with SHP-1. Moreover, SHP-1 formed complexes with GCP3, GCP5, and GCP6 (Supplemental Figure S2B). As expected, Ab to γ-tubulin coprecipitated GCP2 (Figure 3IE, lane 3) and GCP4 (Figure 3II, lane 3), Ab to GCP2 coprecipitated GCP4 (Figure 3IJ, lane 3) and γ-tubulin (Figure 3IN, lane 3), and finally, Ab to GCP4 coprecipitated GCP2 (Figure 3IG, lane 3) and γ-tubulin (Figure 3IO, lane 3).

To independently confirm the interaction of SHP-1 with γ-tubulin, we performed immunoprecipitation experiments using extracts from cells expressing EGFP tagged SHP-1 or EGFP alone. For this, we used HEK cells that could be easily transfected. The antibody to GFP coprecipitated γ-tubulin from HEK cells expressing mSHP-1_EGFP (Supplemental Figure S2C, lane 4) but not from cells expressing EGFP alone (Supplemental Figure S2C, lane 5). The isotype controls for the immunoprecipitation experiments with mouse and rabbit Abs are shown in Supplemental Figure S2D.

The combined data indicate that SHP-1 is capable of forming complexes with γ-TuRC proteins. This finding was further supported by a separation of the extracts from BMMCs on a Superose 6B column. SHP-1 was mainly distributed in low molecular weight fractions, but its part was also detected in high molecular weight pools, where γ-tubulin was present in the form of γTuRCs. On the other hand, the control actin was not detected in the high molecular fractions (Figure 3II).

### 3.2. Characterization of BMMCs Deficient in SHP-1

**Figure 4:**
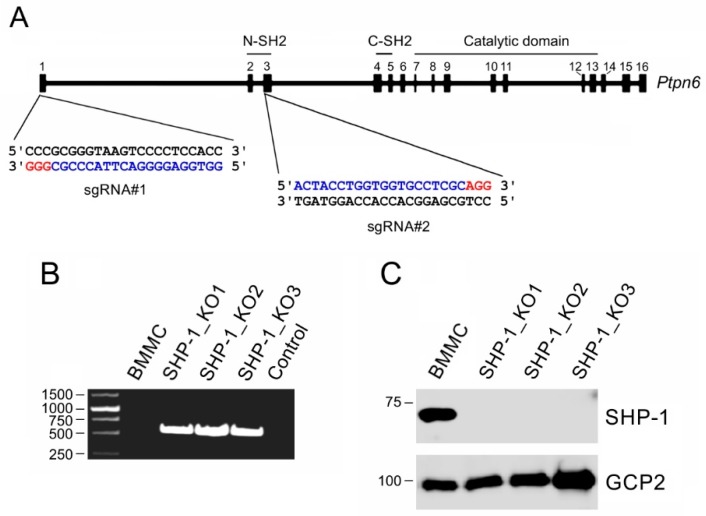
The generation of SHP-1 deficient cell lines. (**A**) A schematic diagram of *Ptpn6* with sites targeted by guide RNA (sgRNA) sequences: The targeted sites (blue) and protospacer-adjacent motifs (PAM; red) on the gene (18.01 kb) containing 16 exons are shown. The defined domains are indicated. (**B**) The PCR amplification of genomic DNA from the control cells (BMMC) and SHP-1-deficient cell lines (SHP-1_KO1, SHP-1_KO2, SHP-1_KO3) with primers flanking the deleted region: The template is not present in the control sample. Due to the large size of the deleted region (approx. 6 kb), no amplification was found in the control BMMCs. The amplification of short fragments (approx. 560 bp) was detected in SHP-1-deficient clones. (**C**) The SHP-1 protein levels in BMMCs and SHP-1-deficient cell lines analyzed by an immunoblotting of whole-cell lysates: GCP2 served as the loading control.

To evaluate the effect of SHP-1 on microtubule nucleation, we prepared BMMC lines lacking SHP-1. For this, we took advantage of CRISP/Cas 9 editing. To delete the gene region containing the canonical and alternative start codons, BMMCs were transfected with CRISPR/Cas9 vectors (sgRNA#1, sgRNA#2, SpCas9) together with reporter plasmid pRR-mSHP1-puro for an enrichment of the cells not expressing SHP-1. A schematic diagram of the mouse SHP-1 gene with sites targeted by sgRNA#1 and sgRNA#2 enabling an efficient depletion of SHP-1 is shown in Figure 4A. We established three independent cell lines (denoted SHP-1_KO1, SHP-1_KO2, and SHP-1_KO3) that have deletions in the targeted region (Figure 4B) and undetectable SHP-1 in immunoblotting (Figure 4C). If not mentioned otherwise, in the following figures, we documented the results with SHP-1_KO1 cells (abbreviated SHP-1_KO).

**Figure 5:**
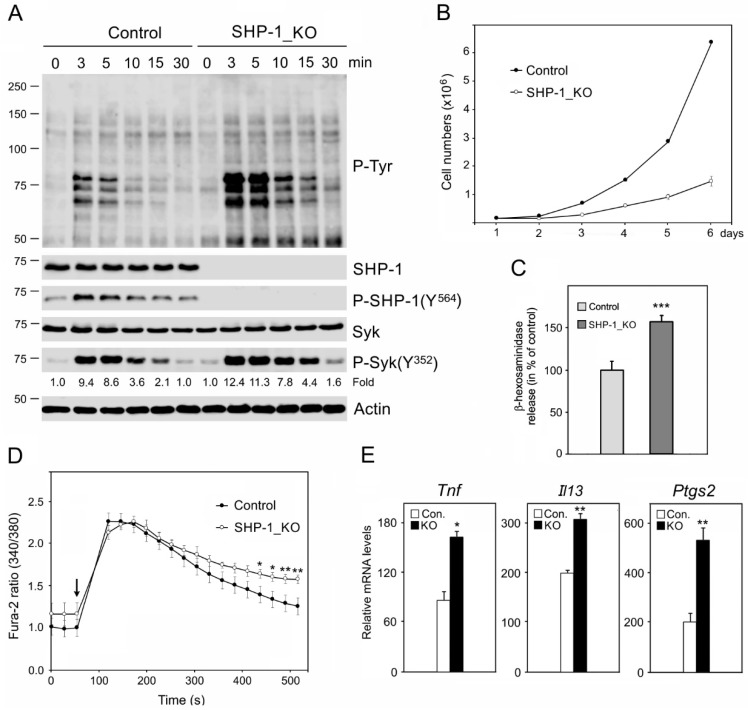
The characterization of cells lacking SHP-1. (**A**) The immunoblot characterization of control BMMCs and BMMCs without SHP-1 (SHP-1_KO): The cells were activated by FcεRI aggregation using an antigen (100 ng/mL) for various time intervals, and the blots from whole-cell lysates were probed with antibodies to phosphotyrosine (P-Tyr), SHP-1, phosphorylated SHP-1 (P-SHP-1[Y^564^]), Syk, and phosphorylated Syk (P-Syk[Y^352^]). Actin served as the loading control. Shown is a representative image out of three repetitions. The numbers under the P-Syk (Y^352^) blot indicate relative amounts of phosphorylated Syk normalized to unstimulated cells and the amount of Syk in the individual samples. (**B**) The growth curves in the control BMMCs and SHP-1_KO cells: A total of 2 × 10^5^ cells was plated in both cell lines. The values indicate the mean ± SD (*n* = 3). (**C**) The degranulation in the control BMMCs and SHP-1_KO cells: The cells were activated by Ag (100 ng/mL), and the degranulation was measured by β-hexosaminidase release. The data represent the mean ± SD (*n* = 3), *** *p* < 0.00001. (**D**) The intracellular Ca^2+^ mobilization during cell activation in the control BMMCs and SHP-1_KO cells: IgE-sensitized cells were loaded with Fura-2-acetoxymethyl ester and activated by a high-affinity IgE receptor aggregation with Ag (100 ng/mL). The arrow indicates the addition of Ag. The data represent the mean ± SD (n = 3) from the independent experiments performed in duplicates; * *p* < 0.05; ** *p* < 0.01. (**E**) The cytokine (TNFα and IL-13) and prostaglandin (COX-2) expressions in the control BMMCs and SHP-1_KO cells in a RT-PCR analysis: The cells were sensitized and either unstimulated or stimulated with Ag (100 ng/mL) for 30 min. The obtained values were normalized with an internal glyceraldehyde-3-phosphate dehydrogenase (GAPDH) control, and the fold increases were determined relative to the unstimulated BMMCs, which was arbitrarily designated a value of 1.0. The data represent the mean ± SEM (*n* = 3); * *p* < 0.05; ** *p* < 0.01.

Compared to the control BMMCs, the absence of SHP-1 resulted in an increase of the P-Tyr protein level during the activation (Figure 5A; P-Tyr). Src family protein tyrosine kinases phosphorylate SHP-1 and the phosphorylation of Y^564^ on SHP-1 are critical to achieving a maximal phosphatase activity [39]. In the course of the control cell activation, a transient increase of the phosphorylation level on Y^564^ of SHP-1 was detected (Figure 5A, P-SHP-1[Y^564^]). SHP-1 negatively regulates the tyrosine phosphorylation of the Syk kinase, which is important for the signal propagation in activated mast cells [3]. The phosphorylation of Syk on Y^352^ releases its autoinhibition and marks active kinase [40,41]. Here, we show that while the deficiency in SHP-1 (SHP-1_KO cells) did not affect the level of Syk (Figure 5A, Syk), the phosphorylation level on Y^352^ of Syk in the course of the activation was significantly higher (Figure 5A, P-Syk [Y^352^]). An evaluation of all the data obtained by densitometry quantification of P-Syk Y^352^ in the control and SHP-1_KO cells is shown in Supplemental Figure S1B. Proliferation was hampered in SHP-1-deficient cells, as demonstrated in the growth curves (Figure 5B). Both the release of β-hexosaminidase (Figure 5C) and the Ca^2+^ influx (Figure 5D) increased in activated SHP-1_KO cells compared with the control BMMCs. The deficiency in SHP-1 also affected the expression of cytokines and prostaglandins. The levels of mRNA for cytokine tumor necrosis factor (TNFα; gene *Tnf*) and interleukin 13 (IL-13; gene *Il13*) increased in SHP-1_KO cells compared to control cells, and the same held true for prostaglandin-endoperoxidase synthase 2 (COX-2; gene *Ptgs2*) mRNA, essential for the production of prostaglandins (Figure 5E). A significantly higher number of cells with microtubule protrusions was observed in the course of Ag-induced activation in the SHP-1_KO cells compared to the controls (Supplemental Figure S3E, SHP-1_KO).

### 3.3. The Absence of SHP-1 Affects Microtubule Regrowth

**Figure 6:**
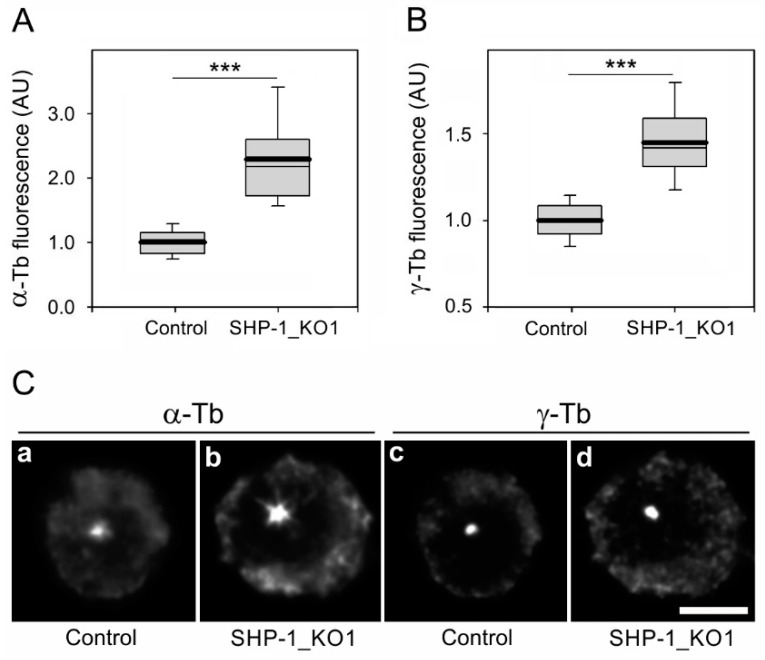
SHP-1 modifies microtubule regrowth. (**A**,**B**) The distribution of α-tubulin or γ-tubulin fluorescence intensities (arbitrary units (AU)) in 1-μm ROI at 1.5 min of regrowth in BMMCs (Control) and SHP-1 deficient BMMCs (SHP-1_KO1) is shown as box plots (three independent experiments, >30 cells counted for each experimental condition). (**A**) The box plot of α-tubulin fluorescence intensities in SHP-1_KO1 cells (*n* = 100) relative to the control cells (Control, *n* = 100). (**B**) The box plot of γ-tubulin fluorescence intensities in SHP-1_KO1 cells (n = 100) relative to the control cells (Control, n = 100). The bold and thin lines within the box represent the mean and median (the 50th percentile), respectively. The bottom and top of the box represent the 25th and 75th percentiles. The whiskers below and above the box indicate the 10th and 90th percentiles. *** *p* < 0.00001. (**C**) The labeling of α-tubulin and γ-tubulin in the microtubule regrowth experiment in the control cells (Control; **a**,**c**) and SHP-1_KO1 cells (**b**,**d**). The cells were fixed with F/Tx/M at 1.5 min of regrowth. The pairs of images Figure 6a,b and Figure 6c,d were collected and processed in exactly the same manner. Scale bar, 5 μm.

The de novo formation of microtubules from interphase centrosomes in BMMCs and SHP-1_KO cells was followed by microtubule regrowth in nocodazole-washout experiments as previously described [16,18]. The extent of microtubule regrowth could be modulated by mechanisms regulating either microtubule nucleation or microtubule dynamics. It has been previously reported that microtubule dynamics is regulated in the cell periphery [42]. Three independent experiments were performed with cells deficient in SHP-1 and control BMMCs. α-Tubulin and γ-tubulin immunofluorescence was measured 1.5 min after a washout in a 1.0-µm ROI. When compared with control BMMCs, an increase in microtubule regrowth was observed in both the SHP-1_KO1 (Figure 6A) and SHP-1_KO2 (Supplemental Figure S3A) cells. The typical staining of α-tubulin in the control and SHP-1_KO1 cells is shown in Figure 6Ca,b. The quantification of γ-tubulin immunofluorescence revealed that the amount of γ-tubulin in centrosomes increased in both SHP-1_KO1 (Figure 6B) and SHP-1_KO2 (Supplemental Figure S3B). In contrast to γ-tubulin, the amount of pericentriolar marker pericentrin [43] was not affected (Supplemental Figure S3C,D). The typical staining of γ-tubulin in the control and SHP-1_KO1 cells is shown in Figure 6Cc,d. The importance of SHP-1 in the modulation of proteins interacting with γ-tubulin during activation events was disclosed by an in vitro kinase assay after precipitation with Ab to γ-tubulin. The phosphorylation of proteins associated with γ-tubulin was higher in SHP-1_KO cells compared to the control BMMCs (Supplemental Figure S3G, 3 min).

**Figure 7:**
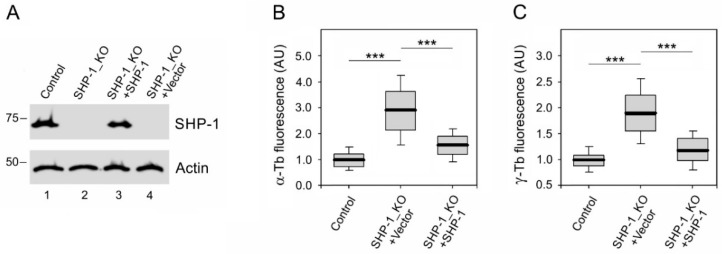
Microtubule regrowth in a phenotypic rescue experiment. (**A**) An immunoblot analysis of SHP-1 in whole-cell lysates from BMMCs (Control), SHP-1_KO cells, SHP-1_KO cells rescued by mSHP-1 in lentiviral vector (SHP-1_KO+SHP-1), and SHP-1_KO cells infected by empty vector (SHP-1_KO+Vector): The blots were probed by Abs to SHP-1 and Actin (loading control). (**B**,**C**) The distribution of α-tubulin or γ-tubulin fluorescence intensities (arbitrary units (AU)) in 1-μm ROI at 1.5 min of regrowth in BMMCs (Control), SHP-1_KO cells infected by empty vector (SHP-1_KO+Vector), and SHP-1_KO cells rescued by mSHP-1 in lentiviral vector (SHP-1_KO+SHP-1) is shown as box plots (three independent experiments, >50 cells counted for each experimental condition). (**B**) The box plot of α-tubulin fluorescence intensities in SHP-1_KO+Vector (*n* = 159) and SHP-1_KO+SHP-1 cells (*n* = 236) relative to the control cells (Control, n = 315). (**C**) The box plot of γ-tubulin fluorescence intensities in SHP-1_KO+Vector (*n* = 255) and SHP-1_KO+SHP-1 cells (*n* = 244) relative to the control cells (Control, *n* = 315). The bold and thin lines within the box represent the mean and median (the 50th percentile), respectively. The bottom and top of the box represent the 25th and 75th percentiles. The whiskers below and above the box indicate the 10th and 90th percentiles. *** *p* < 0.00001.

For the phenotypic rescue experiments, we prepared a lentiviral vector encoding mouse SHP-1. To rescue microtubule regrowth, SHP-1 in the vector or empty vector were expressed in SHP-1_KO cells. A typical result of an immunoblotting experiment is shown in Figure 7A. When compared with the control BMMCs, an increase in the microtubule regrowth was observed in SHP-1_KO cells with the empty vector, while the introduction of SHP-1 into these cells restored microtubule regrowth to that in control cells (Figure 7B). The quantification of γ-tubulin immunofluorescence revealed that the amount of γ-tubulin in centrosomes also increased in SHP-1_KO cells with the empty vector and was restored after the introduction of SHP-1 (Figure 7B). An introduction of SHP-1 into deficient cells also restored degranulation (Supplemental Figure S3F).

Altogether, these data suggest that SHP-1 negatively regulates microtubule nucleation from the centrosomes, and its regulatory role is conveyed by the amount of γ-tubulin/γTuRCs.

### 3.4. Inhibition of Enzymatic Activity of SHP-1 Modulates Microtubule Regrowth

**Figure 8:**
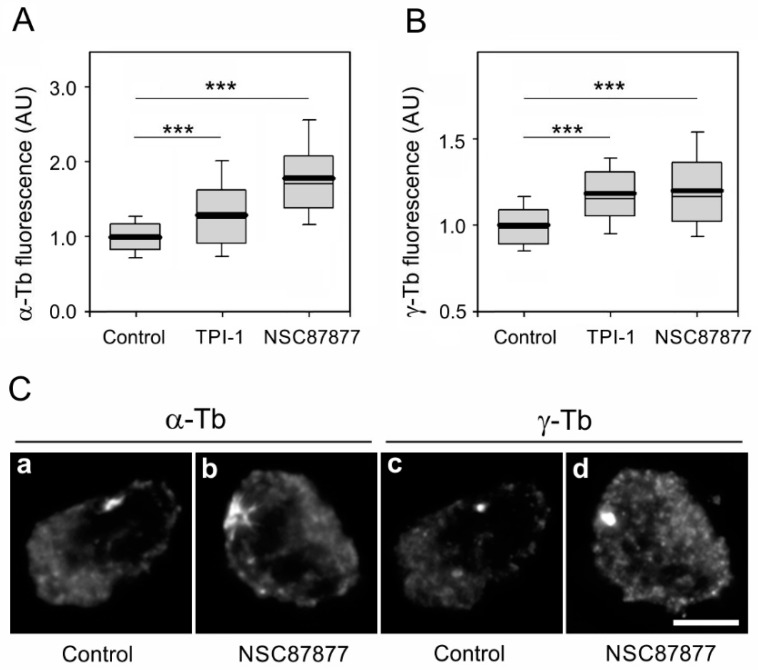
The inhibition of SHP-1 activity augments microtubule nucleation. (**A**,**B**) BMMCs were cultivated with 100 nM TPI-1, 500 nM NSC87877, or a DMSO carrier (Control) for 1 h before and during the microtubule regrowth assay. The distribution of α-tubulin or γ-tubulin fluorescence intensities (arbitrary units (AU)) in 1-μm ROI at 1.5 min of regrowth is shown as box plots (three independent experiments, >50 cells counted for each experimental condition). (**A**) The box plot of α-tubulin fluorescence intensities in TPI-1 (*n* = 158) or NSC87877 (*n* = 158) preincubated cells relative to the control cells (Control, n = 158). (**B**) The box plot of γ-tubulin fluorescence intensities in TPI-1 (*n* = 158) or NSC87877 (n = 158) preincubated cells relative to the control cells (Control, *n* = 158). The bold and thin lines within the box represent the mean and median (the 50th percentile), respectively. The bottom and top of the box represent the 25th and 75th percentiles. The whiskers below and above the box indicate the 10th and 90th percentiles. *** *p* < 0.00001. (**C**) The labeling of α-tubulin and γ-tubulin in the microtubule regrowth experiment in control cells with a DMSO carrier (Control; **a**,**c**) and cells pretreated with 500 nM NSC87877 for 1 h before and during the microtubule regrowth assay (**b**,**d**). The cells were fixed with F/Tx/M at 1.5 min of regrowth. The pairs of images Figure 8a,b and Figure 8c,d were collected and processed in exactly the same manner. Scale bar, 5 μm.

To find out whether the SHP-1 enzymatic activity is essential for the regulatory role of phosphatase in microtubule nucleation, we performed microtubule regrowth experiments with SHP inhibitors. We used both a highly specific inhibitor TPI-1 at low concentrations, targeting only SHP-1 [44], and a less specific inhibitor NSC87877, targeting both SHP-1 and SHP-2 [45]. The cells were preincubated in the presence of 100 nM TPI-1, 500 nM NSC87877, or a DMSO carrier (Control) for 1 h before the nocodazole washout assay, and the inhibitors were present throughout the assay. The inhibition of phosphatase activity by both inhibitors resulted in an increase of the immunofluorescence signal for both α-tubulin (Figure 8A) and γ-tubulin (Figure 8B). The typical co-staining of α-tubulin and γ-tubulin in the control and NSC87877-treated cells is shown in Figure 8C. These results document that enzymatically active SHP-1 modulates the nucleation of microtubules in BMMCs.

Although SHP-1 is mainly expressed in hematopoietic cells, it is also detected in some epithelial cells, e.g., human epithelial-like breast adenocarcinomas. This is demonstrated by an immunoblotting of the cell extracts from the MCF7 cell line (Supplemental Figure S4A). Precipitation experiments with MCF7 extracts showed that SHP-1 interacted with γ-tubulin, GCP2, and GCP-4 (Supplemental Figure S4A, left panel, IP: SHP-1 lane 3). A reciprocal precipitation with Ab to γ-tubulin confirmed the interaction of SHP-1 with γ-tubulin (Supplemental Figure S4A, right panel, lane 3). Altogether, these experiments suggest that the formation of complexes containing SHP-1 and γ-tubulin complex proteins is not limited to BMMCs. To evaluate whether an inhibition of the SHP-1 activity modulates microtubule regrowth in MCF7 cells, we performed microtubule regrowth assay in cells preincubated with TPI-1. Similarly, as in BMMCs, an inhibition of the SHP-1 activity resulted in an enhancement of microtubule regrowth (Supplemental Figure S4B) and the accumulation of γ-tubulin in centrosomes (Supplemental Figure S4C). These data suggest that active SHP-1 can regulate microtubule nucleation, by affecting the centrosomal accumulation of γ-tubulin in various cell types.

### 3.5. SHP-1 Interacts with Syk Protein Tyrosine Kinase Associating with γ-Tubulin Complex Proteins

Even though SHP-1 affected microtubule nucleation from centrosomes in BMMCs, we failed to localize the phosphatase to a centrosomal region, using a limited number of commercial Abs, both in resting cells and cells activated by FcεRI aggregation. On the other hand, SHP-1 regulated the activity of the Syk kinase (Figure 5), which was reported to interact with γ-tubulin [6]. Moreover, in breast cancer cells, Syk localized to centrosomes [46,47]. We, therefore, investigated whether, in BMMCs, Syk forms complexes with SHP-1 and localizes to centrosomes.

**Figure 9:**
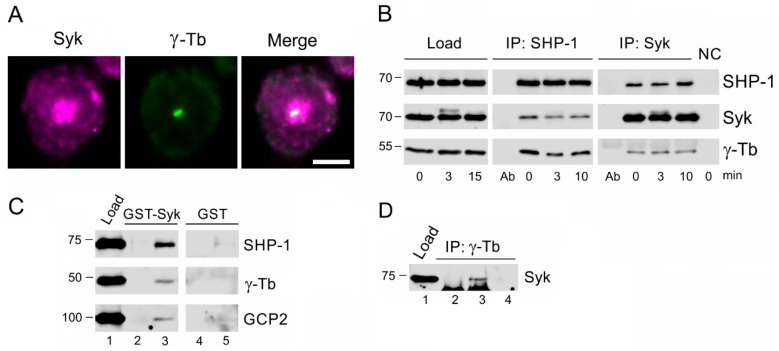
Syk kinase localizes to a centrosomal region and associates both with γ-tubulin complex proteins and SHP-1. (**A**) The double-label immunofluorescence staining of Syk and γ-tubulin: The cells were fixed with F/Tx/M and stained for Syk (**a**) in magenta and for γ-tubulin (**b**) in green. A superimposition of the images (Figure 9a,b) is also shown (**c**). Scale bar, 5 µm. (**B**) The immunoprecipitation experiments: Extracts from the nonactivated (0 min) or antigen-activated (3 min, 10 min) BMMCs were precipitated with the immobilized Abs specific to SHP-1 or Syk. The blots were probed with Abs to SHP-1, Syk, and γ-tubulin (γ-Tb). Ab, immobilized Ab not incubated with cell extract; NC, carrier without Ab, incubated with cell extract (negative control). (**C**) The pull-down assay: Extracts from BMMCs incubated with the GST-tagged whole-length Syk or GST alone were immobilized on glutathione-Sepharose beads. The blots of the bound proteins were probed with abs to SHP-1, γ-Tb, and GCP2. The load (*lane 1*), the immobilized GST-Syk not incubated with cell extracts (*lane 2*), the proteins bound to GST-Syk (*lane 3*), the immobilized GST alone not incubated with cell extracts (*lane 4*), and the proteins bound to GST alone (*lane 5*). (**D**) The immunoprecipitation experiment: Extracts from BMMCs were precipitated with immobilized Ab specific to γ-tubulin. The blot was probed with Ab to Syk. The load (*lane 1*), the immobilized Ab not incubated with cell extracts (*lane 2*), the precipitated proteins (*lane 3*), and the carrier without Ab and incubated with cell extracts (*lane 4*).

Double-label immunofluorescence experiments with Abs to Syk and γ-tubulin revealed that, in BMMCs, Syk accumulated in a broad pericentrosomal region (Figure 9A, Syk), where centrosomal γ-tubulin was located (Figure 9A, γ-Tb). SHP-1 precipitated in the course of the activation Syk and γ-tubulin (Figure 9B, IP: SHP-1), and a reciprocal precipitation with Ab to Syk (Figure 9B, IP: Syk) corroborated the interaction of Syk with SHP-1 and γ-tubulin. SHP-1 precipitated less Syk in activated cells when compared to resting cells. Pull-down experiments with GST-tagged whole-length Syk confirmed that, in BMMCs, SHP-1, γ-tubulin, and GCP-2 associate with recombinant Syk (Figure 9C, lane 3) but not with GST alone (Figure 9C, lane 5). Immunocomplexes containing γ-tubulin and Syk were also detected when the antibody to γ-tubulin was used for precipitation (Figure 9D, lane 3). The activities of Syk and associated proteins during activation were modulated by SHP-1 as disclosed in vitro kinase assays after a precipitation with Ab to Syk. The phosphorylation of proteins associated with Syk was higher in SHP-1_KO cells compared to the control BMMCs (Supplemental Figure S3H).

Collectively, these results suggest that SHP-1 is capable of forming complexes with Syk that interact with γ-tubulin complex proteins. Consequently, the regulatory role of SHP-1 on microtubule nucleation might be due to a modulation of the Syk activity.

## 4. Discussion

The Ag-induced activation of mast cells leads to rapid cytoskeleton rearrangements and degranulation. Accumulated data point to the importance of microtubules in this process [5,8,9,10,48]. We have shown previously that the stimulation of mast cells by FcεRI aggregation triggers the generation of complexes containing γ-tubulin, tyrosine-phosphorylated proteins, and tyrosine kinases [6,49]; a transient increase in the amount of polymerized tubulin [6]; and a reorganization of the microtubules [6,7,50]. The opposing actions of protein tyrosine kinases and protein tyrosine phosphatases determine the level of tyrosine phosphorylation during the activation events. It is well-accepted that protein tyrosine kinases are essential during mast cell signaling, but the exact function of protein tyrosine phosphatases is less understood. Here, we report on SHP-1 tyrosine phosphatase interacting with proteins of γ-tubulin complexes and modulating microtubule nucleation from the centrosomes of BMMCs. SHP-1 represents a negative regulator of microtubule nucleation. Our study provides a possible mechanism for the concerted action of tyrosine kinases and tyrosine phosphatases in the regulation of microtubule formation in activated mast cells.

Several lines of evidence indicate that the association of SHP-1 with γ-tubulin complex proteins is specific. First, reciprocal precipitation experiments confirmed an interaction between SHP-1 and proteins constituting γ-TuRCs. Second, two SHPs (SHP-1 and SHP-2) are expressed in BMMCs, but only SHP-1 interacted with the γ-tubulin complex proteins. Third, GFP-tagged SHP-1 interacted with γ-tubulin. Fourth, a gel filtration chromatography revealed that the high molecular weight pool of SHP-1 co-distributed with γ-TuRCs. Finally, the absence of SHP-1 in SHP-1_KO cells increased the phosphorylation of proteins in γ-tubulin immunocomplexes. Interestingly, such an association was not limited to BMMCs, as SHP-1 was also present in γ-tubulin immunocomplexes prepared from human epithelial breast cells MCF7. These findings suggest that the direct or indirect interactions between SHP-1 and γ-tubulin complex proteins might occur in various cell types.

Microtubule nucleation at the centrosome occurs from γ-TuRCs located in the pericentriolar material [13]. We, therefore, asked whether SHP-1 regulates microtubule nucleation in BMMCs by affecting the centrosomal γ-tubulin levels. Our data from measuring the γ-tubulin signal in regrowth experiments from cells lacking SHP-1 or cells with an inhibited SHP-1 enzymatic activity suggest that SHP-1 prevents γ-tubulin accumulation at the centrosome. Moreover, this SHP-1 function is not limited to BMMCs, as it was also found in epithelial MCF7 cells expressing SHP-1. On the other hand, no changes in the amount of pericentrin were detected, indicating that the pericentriolar matrix integrity was not affected. Although SHP-1 represents a negative regulator of microtubule nucleation, we did not locate SHP-1 into the centrosome using a limited number of commercial Abs. However, we cannot rule out that SHP-1 associates with the centrosomes only transiently. Alternatively, it could modulate cytosolic proteins before their interaction(s) with centrosomes. In principle, SHP-1 could affect tyrosine-phosphorylated γTuRCs proteins or tyrosine-phosphorylated proteins that target, anchor, or activate γ-TuRCs [43]. Alternatively, SHP-1 could regulate protein kinases modulated by tyrosine.

In contrast to αβ-tubulin dimers [51,52], the posttranslational modification of γ-tubulin is less prominent [53]. However, it has been repeatedly reported that γ-tubulin is phosphorylated [14,30,54]. The phosphorylation of γ-tubulin residue Tyr 445, which is invariably present in all γ-tubulins, was described, and a mutation of this residue changed the microtubule dynamics [14]. However, the precipitation of γ-tubulin from resting or activated BMMCs, followed by immunoblotting, did not reveal its phosphorylation on tyrosine [6]. Proteomic studies revealed the tyrosine phosphorylation sites on human GCP2, GCP3, GCP5, and GCP6 [55]. Collectively, these data suggest that tyrosine kinases could regulate γ-tubulin properties. Because GCPs coordinate the arrangement of γ-tubulin in γ-TuRCs, the phosphorylation of GCPs could also regulate the conformational changes that might be required for γ-TuRC activation [56].

Evidence suggests an important role for tyrosine kinases in the regulation of microtubule organization from centrosomes. The Fyn kinase was found on centrosomes in myelocytic leukemia cells HL-60 [57] and human T-lymphocytes [58]. The JAK2 tyrosine kinase specifically associates with centrioles and regulates microtubule anchoring [59]. Androgen and Src signaling modulated the microtubule nucleation during interphase by promoting the centrosomal localization of γ-tubulin [16] via the activation of the MAPK/Erk signaling pathway [17]. It has also been shown that Syk is catalytically active at the centrosome [46,47]. In the early stages of BMMCs activation, when microtubule formation is stimulated, tyrosine-phosphorylated proteins concentrate in the centrosomal region. Inhibitors of Src or Syk kinases inhibited the phosphorylation of proteins interacting with γ-tubulin in activated BMMCs [6]. An association of Src family kinases with γ-tubulin complexes was also reported in activated rat basophilic leukemia cell line RBL-2H3 [49] and in differentiating mouse P19 embryonic carcinoma cells [30,60].

Although several protein tyrosine phosphatases co-localize with γ-tubulin on the centrosome, e.g., PTB-BL (PTPN13) [61] or PRL-1 (PTP4A1) [62], our knowledge of their involvement in the regulation of microtubule nucleation from centrosomes is very limited. In the case of dual-specificity phosphatase CDC25B, essential for the regulation of the cell cycle, it was reported that the inhibition of its activity suppressed an assembly of interphase microtubules and the centrosomal localization of γ-tubulin [63]. This indicates that different protein tyrosine phosphatases might be involved in distinct signaling pathways with respect to the regulation of microtubule nucleation. SHP-1 in BMMCs might balance the stimulating effect of the Src and Syk/ZAP families on microtubule formation.

The signaling pathway leading to an inhibition of microtubule nucleation via the activation of SHP-1 is currently unknown. Interestingly, multidomain G protein-coupled receptor kinase-interacting protein 1 (GIT1) [64] and p21-activated kinase interacting exchange factor (βPIX) [65] are substrates for tyrosine kinases and associate with centrosomes of fibroblasts [66]. Previously, we have shown that, in BMMCs, these signaling proteins also located at the centrosomes and formed complexes with γ-tubulin and the Ag-induced cell activation stimulates their phosphorylation on tyrosines [18]. Moreover, γ-tubulin binds directly to the N-terminal ARF GTPase-activating protein (ARF-GAP) domain (aa 1–124) of GIT1 in vitro [33]. GIT1 has been shown to be phosphorylated in cells in a Src kinase-dependent manner [67], and different studies have pointed to the relevance of tyrosine phosphorylation in the regulation of GIT1 functions. It has been shown that the tyrosine phosphorylation of GIT1 is required for intramolecular conformational changes in GIT1 and the release of its auto-inhibitory interaction [68]. The tyrosine phosphorylation of GIT1 in stimulated mast cells might, thus, lead to its activation. On the other hand, the tyrosine phosphorylation of βPIX, in a Src kinase-dependent manner weakens its ability to bind GIT1 [69]. We have shown that GIT1 and βPIX regulate microtubule nucleation from interphase centrosomes in various cell types [18,33]. We have found that the tyrosine phosphorylation level on GIT1 increased during BMMCs activation and was higher in SHP-1_KO cells. On the other hand, we did not detect the tyrosine phosphorylation of βPIX (Supplemental Figure S3I). SHP-1 could, thus, affect activated BMMC in both the activity of Syk kinase and the conformational state of GIT1. Deciphering the role of SHP-1 in the modulation of GIT1/βPIX complexes warrants further investigation. Other proteins essential for microtubule nucleation might be potentially modulated by SHP-1. Proteomic studies revealed that the tyrosine phosphorylation of proteins is important for targeting or anchoring γ-TuRCs to centrosomes; e.g., CDK5RAP2, NEDD1, ninein, and pericentrin [70].

An increased degranulation was reported in BMMCs isolated from mew mice deficient in SHP-1 [71]. On the other hand, a decreased degranulation was found in another study using BMMC from SHP-1 deficient me mice [72]. The discrepancy between studies was explained by different experimental setups [71]. In this study, the increased microtubule nucleation in established BMMC line lacking SHP-1 correlated with an increased degranulation. The degranulation returned to the level in wild-type cells when SHP-1 was introduced into the deficient cells. Our data, thus, suggest that SHP-1 negatively regulates Ag-induced degranulation.

In conclusion, our data suggest a novel mechanism of microtubule modulation in mast cells with SHP-1 tyrosine phosphatase as a negative regulator of microtubule nucleation. Presumably, also through this action, SHP-1 is involved in the spatiotemporal regulation of degranulation. An interference with the microtubular network via specific regulators of microtubule nucleation in mast cells could open up new rational approaches to the treatment of inflammatory and allergic diseases.

## Supplementary Materials

The following are available online at https://www.mdpi.com/2073-4409/8/4/345/s1, Text S1: Features of the in-house written macro for processing images from the microtubule regrowth experiment, Table S1: The sequences of the primers used for the RT-qPCR analysis of mouse genes, Figure S1: The quantitative analysis of the overall tyrosine phosphorylation and tyrosine phosphorylation of Syk in the course of BMMC activation, Figure S2: The association of SHP-1 with γ-tubulin complex proteins and the isotype controls for the immunoprecipitation experiments, Figure S3: The characterization of SHP-1-deficient BMMCs and microtubule regrowth, Figure S4: SHP-1 modifies the microtubule regrowth and associates with γ-tubulin complex proteins in epithelial MCF7 cells.

## Abbreviations

Ab(s)	Antibody(ies)
Ag	Antigen
BMMC(s)	Bone marrow-derived mast cell(s)
GCP	γ-tubulin complex protein
mAb	Monoclonal antibody
P-Tyr	Phosphotyrosine
ROI	Region of interest
γTuRC	γ-tubulin ring complex
γTuSC	γ-tubulin small complex
