# Supplementary material for: Regulation of Microtubule Nucleation in Mouse Bone Marrow-Derived Mast Cells by Protein Tyrosine Phosphatase SHP-1
Source: Cells. 2019 Apr 11;8(4):345. doi: 10.3390/cells8040345 (PMC6523986; doi:10.3390/cells8040345)
Supplement: Supplementary file 1 [file cells-08-00345-s001.zip › Figure S1.pdf]

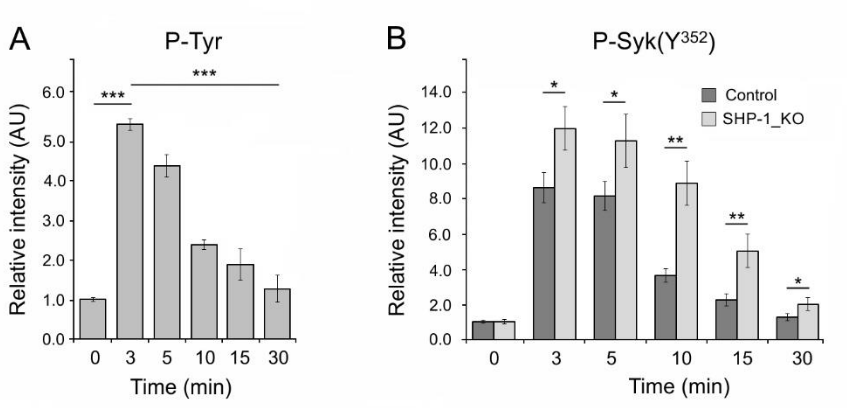


**Figure S1.** Quantitative analysis of overall tyrosine phosphorylation and tyrosine phosphorylation of Syk in the course of BMMCs activation. **(A)** Densitometric quantification of overall protein tyrosine phosphorylation level shown in Figure 1A. Relative intensities of P-Tyr normalized to non-activated cells and the amount of actin in individual samples. **(B)** Densitometric quantification of Syk kinase $Y^{352}$ phosphorylation level shown in Figure 5A. Relative intensities of P-Syk($Y^{352}$) in control BMMCs and SHP-1_KO cells normalized to non-activated cells and the amount of actin in individual samples. (A-B) Values indicate mean ± SD (n=3), *$p < 0.05$, **$p < 0.01$, ***$p < 0.001$.
