# Supplementary material for: Regulation of Microtubule Nucleation in Mouse Bone Marrow-Derived Mast Cells by Protein Tyrosine Phosphatase SHP-1
Source: Cells. 2019 Apr 11;8(4):345. doi: 10.3390/cells8040345 (PMC6523986; doi:10.3390/cells8040345)
Supplement: Supplementary file 1 [file cells-08-00345-s001.zip › Figure S2.pdf]

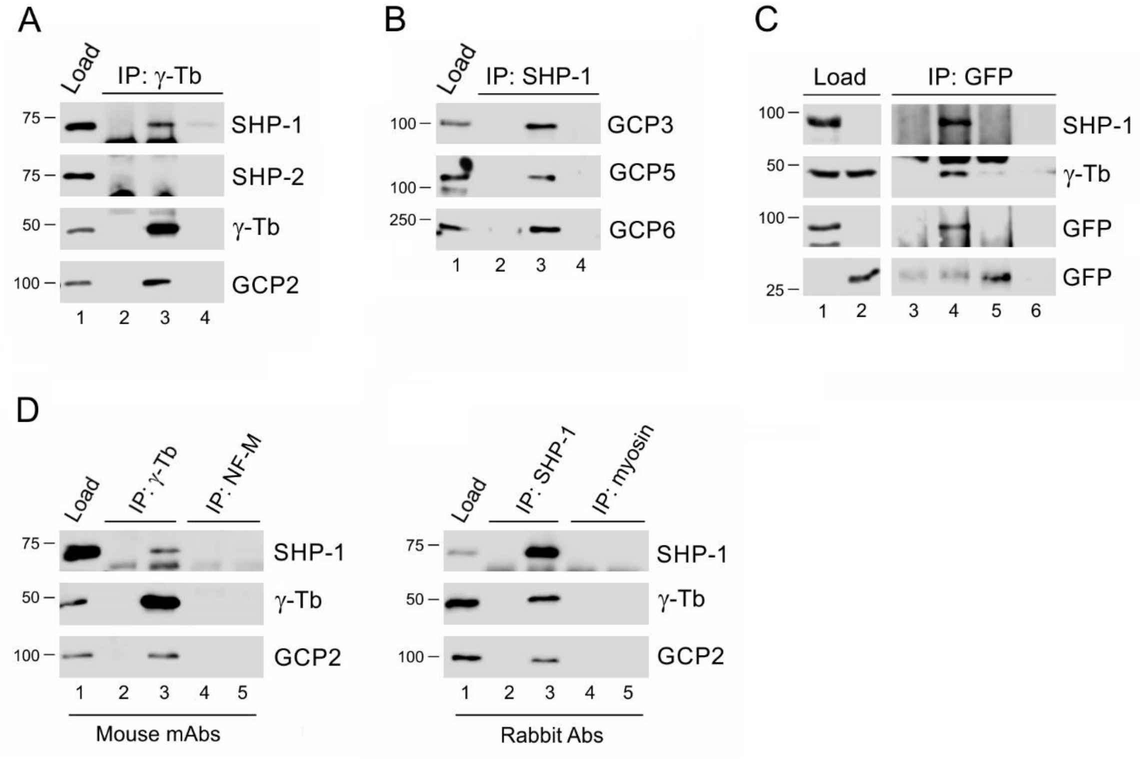

Figure S2. Association of SHP-1 with γ-tubulin complex proteins, and isotype controls for immunoprecipitation experiments. Immunoprecipitation from BMMCs (**A-B, D**) or HEK (**C**) extracts. **(A)** Precipitation with immobilized Ab to γ-tubulin sequence 38-53. Blots probed with Abs to SHP-1, SHP-2, γ-tubulin (γ-Tb) and GCP2. Load (*lane 1*), immobilized Ab not incubated with cell extract (*lane 2*), precipitated proteins (*lane 3*), and carrier without Ab, incubated with cell extracts (*lane 4*). **(B)** Precipitation with immobilized Ab to SHP-1. Blots probed with Abs to GCP3, GCP5 and GCP6. Load (*lane 1*), immobilized Ab not incubated with cell extract (*lane 2*), precipitated proteins (*lane 3*), and carrier without Ab, incubated with cell extracts (*lane 4*). **(C)** Precipitation with immobilized Ab to GFP. Blots probed with Abs to SHP-1, γ-tubulin (γ-Tb) and GFP. Load, HEK cells expressing SHP-1_EGFP (*lane 1*), Load, cells expressing EGFP (*lane 2*), immobilized Ab not incubated with cell extract (*lane 3*), precipitated proteins from cells expressing SHP-1_EGFP (*lane 4*), cells expressing EGFP (*lane 5*), and carrier without Ab, incubated with cell SHP-1_EGFP cell extract (*lane 6*). **(D)** Isotype controls for immunoprecipitation experiments. Extracts were precipitated with immobilized mouse mAb to γ-tubulin (IgG2b), mouse mAb to NF-M (IgG2a), rabbit Ab to SHP-1, or rabbit Ab to myosin. Blots were probed with Abs to SHP-1, γ-tubulin (γ-Tb) and GCP2. Load (*lane 1*), immobilized Abs not incubated with cell extracts (*lanes 2 and 4*), precipitated proteins (*lanes 3 and 5*).
