# Supplementary material for: Regulation of Microtubule Nucleation in Mouse Bone Marrow-Derived Mast Cells by Protein Tyrosine Phosphatase SHP-1
Source: Cells. 2019 Apr 11;8(4):345. doi: 10.3390/cells8040345 (PMC6523986; doi:10.3390/cells8040345)
Supplement: Supplementary file 1 [file cells-08-00345-s001.zip › Figure S3-revision.pdf]

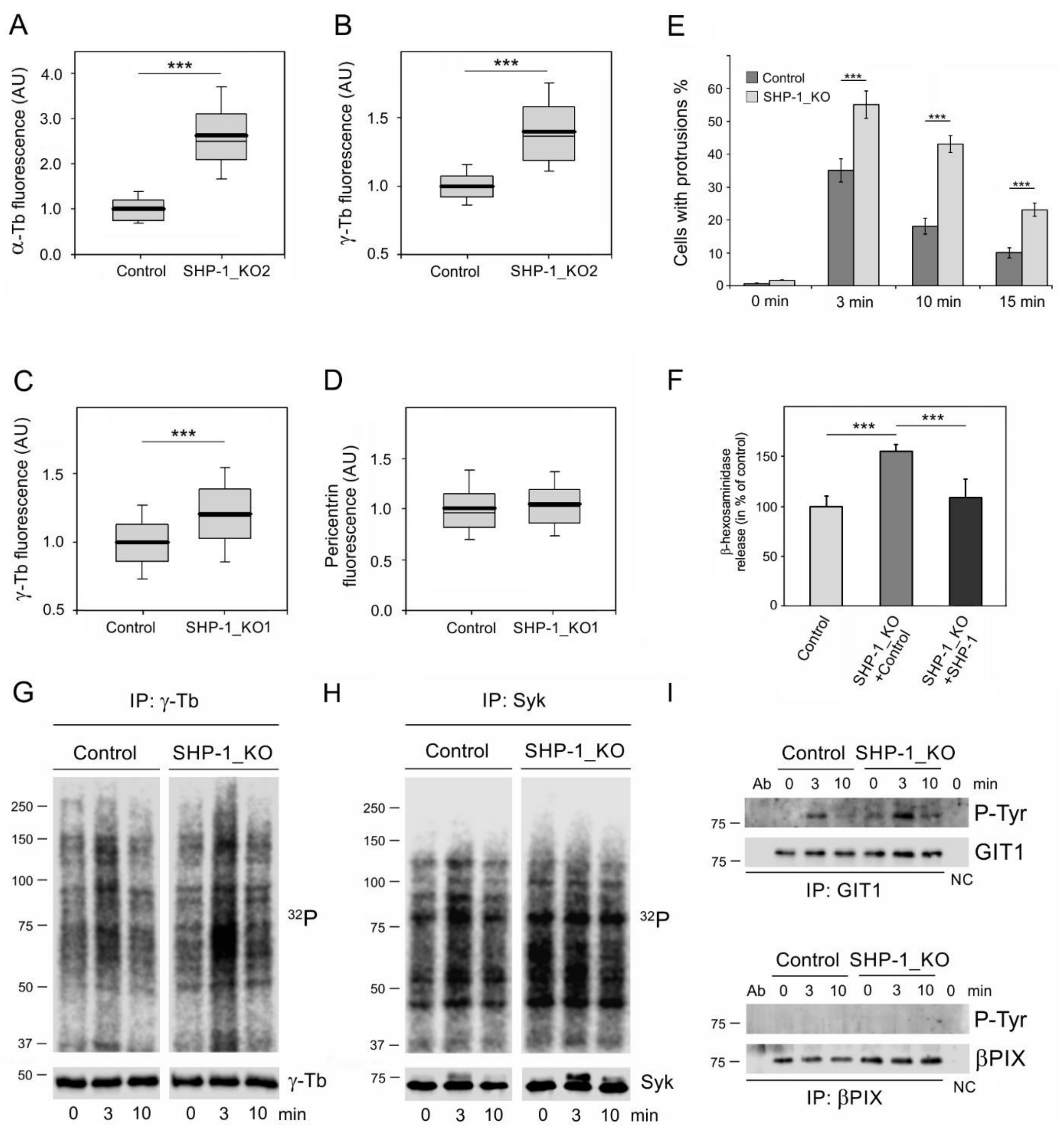

Figure S3. Characterization of SHP-1 deficient BMMCs, and microtubule regrowth. (**A-B**) Distribution of α-tubulin or γ-tubulin fluorescence intensities (arbitrary units [AU]) in 1-μm ROI at 1.5 min of regrowth in BMMCs (Control) and SHP-1 deficient cells (SHP-1_KO2) is shown as box plots (three independent experiments, > 50 cells counted for each experimental condition). (**A**) Box plot of α-tubulin fluorescence intensities in SHP-1_KO2 cells (n = 155) relative to control cells (Control, n = 155). (**B**) Box plot of γ-tubulin fluorescence intensities in SHP-1_KO2 cells (n = 154) relative to control cells (Control, n = 154). Bold and thin lines within the box represent mean and median (the 50th percentile), respectively. The bottom and top of the box represent the 25th and 75th percentiles. Whiskers below and above the box indicate the 10th and 90th percentiles. ***, $p < 1 \times 10^{-5}$. (**C-D**) Distribution of γ-tubulin or pericentrin fluorescence intensities (arbitrary units [AU]) in 1-μm ROI at 1.5 min of regrowth in BMMCs (Control) and SHP-1 deficient cells (SHP-1_KO1) is shown as box plots (three independent experiments, > 80 cells counted for each experimental condition). (**C**)

Box plot of γ-tubulin fluorescence intensities in SHP-1_KO1 cells (n = 248) relative to control cells (Control, n = 272). (**D**) Box plot of pericentrin fluorescence intensities in SHP-1_KO1 cells (n = 225) relative to control cells (Control, n = 272). Bold and thin lines within the box represent mean and median (the 50th percentile), respectively. The bottom and top of the box represent the 25th and 75th percentiles. Whiskers below and above the box indicate the 10th and 90th percentiles. ***, $p < 1 \times 10^{-5}$. **(E)** Quantitative analysis of number of cells with microtubule protrusions in the course of activation in control BMMCs and SHP-1_KO cells. Values indicate mean ± SD (n=3), ****p* < 0.001. **(F)** Degranulation in control BMMCs, SHP-1_KO cells infected by empty lentiviral vector (SHP-1_KO+Vector) and SHP-1_KO cells rescued by mSHP-1 in lentiviral vector (SHP-1_KO+SHP-1). Cells were activated by Ag (100 ng/ml) and degranulation was measure by β-hexosaminidase release. Data represent the mean ± SD (n=3), ***$p < 1 \times 10^{-5}$. (**G-H**) The kinase activity in γ-tubulin and Syk immunocomplexes. Non-activated (0 min) or antigen-activated (3 min, 10 min) control BMMCs and SHP-1_KO cells were precipitated with Abs to γ-tubulin (G) or Syk (H). Immunocomplexes were subjected to *in vitro* kinase assay, electrophoretically separated, and detected by autoradiography (^{32}P). (**I**) Tyrosine phosphorylation of GIT1 and βPIX in control and SHP-1_KO cells during activation by FcεRI aggregation. Extracts from BMMCs precipitated with immobilized Abs specific to GIT1 or βPIX. Blots were first probed with mouse Ab to phosphotyrosine (P-Tyr). The same blots were after that probed with Abs to GIT1 or βPIX, respectively. Ab, immobilized Abs not incubated with cell extract; NC, negative control, carriers without Ab, incubated with cell extracts.
