# Supplementary material for: Regulation of Microtubule Nucleation in Mouse Bone Marrow-Derived Mast Cells by Protein Tyrosine Phosphatase SHP-1
Source: Cells. 2019 Apr 11;8(4):345. doi: 10.3390/cells8040345 (PMC6523986; doi:10.3390/cells8040345)
Supplement: Supplementary file 1 [file cells-08-00345-s001.zip › Figure S4.pdf]

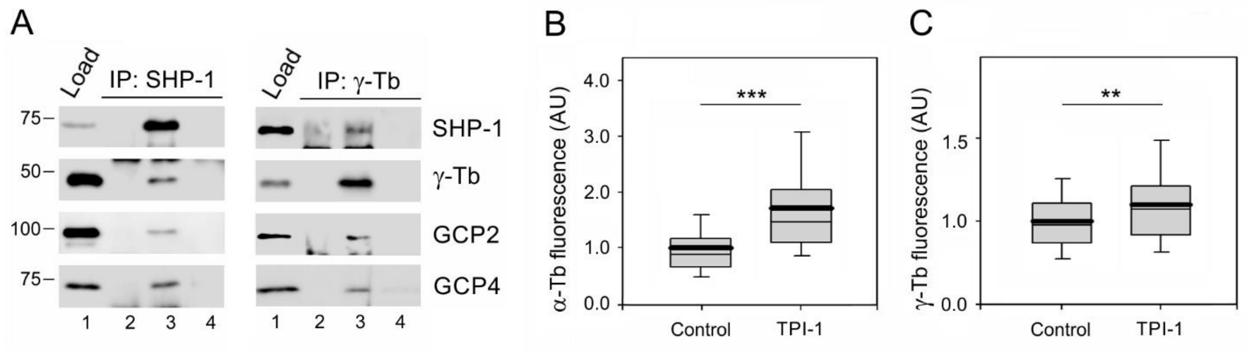

Figure S4. SHP-1 modifies microtubule regrowth, and associates both with γ-tubulin complex proteins in epithelial MCF7 cells. **(A)** Immunoprecipitation experiments. Extracts from MCF7 cells precipitated with immobilized Abs specific to γ-tubulin or SHP-1. Blots probed with Abs to SHP-1, γ-tubulin (γ-Tb), GCP2 and GCP4. Load (*lane 1*), immobilized Abs not incubated with cell extracts (*lane 2*), precipitated proteins (*lane 3*), and carriers without Abs, incubated with cell extracts (*lane 4*). **(B-C)** Cells were cultivated with 100 nM TPI-1 or DMSO carrier (Control) for 1 h before and during microtubule regrowth assay. Distribution of α-tubulin or γ-tubulin fluorescence intensities (arbitrary units [AU]) in 1-μm ROI at 1.5 min of regrowth is shown as box plots (three independent experiments, > 30 cells counted for each experimental condition). **(B)** Box plot of α-tubulin fluorescence intensities in TPI-1 (n = 108) pre-incubated cells relative to control cells (Control, n = 143). **(C)** Box plot of γ-tubulin fluorescence intensities in TPI-1 (n = 90) pre-incubated cells relative to control cells (Control, n = 142). Bold and thin lines within the box represent mean and median (the 50th percentile), respectively. The bottom and top of the box represent the 25th and 75th percentiles. Whiskers below and above the box indicate the 10th and 90th percentiles. **, $p < 0.001$; ***, $p < 1 \times 10^{-5}$.
