# Supplementary material for: Regulation of Microtubule Nucleation in Mouse Bone Marrow-Derived Mast Cells by Protein Tyrosine Phosphatase SHP-1
Source: Cells. 2019 Apr 11;8(4):345. doi: 10.3390/cells8040345 (PMC6523986; doi:10.3390/cells8040345)
Supplement: Supplementary file 1 [file cells-08-00345-s001.zip › Table S1.pdf]

Table S1 Sequences of primers used for RT-qPCR analysis of mouse genes

Name	Sequence	Amplicon length
Ptpn6, fwd	5‘- GAACATTCTTCCCTTTGACC-3’	127 bp
Ptpn6, rev	5‘- TCTTAGAGTTCTCATCTGGAC-3’	
Ptpn11, rev	5’- AACATCACTGGTGTGGAGGC-3’	287 bp
Ptpn11, rev	5’ – TGCACAGTTCAGCGGGTA-3’	
Il13, fwd	5’- CTTAAGGAGCTTATTGAGGAG-3’	144 bp
Il13, rev	5’- CATTGCAATTGGAGATGTTG-3’	
Tnf, fwd	5’- CTATGTCTCAGCCTCTTCTC-3’	108 bp
Tnf, rev	5’- CATTTGGGAACTTCTCATCC-3’	
Ptgs2, fwd	5’- ACTCATAGGAGAGACTATCAAG-3’	147 bp
Ptgs2, rev	5’- GAGTGTGTTGAATTCAGAGG-3’	
Gapdh, fwd	5‘- AACTTTGGCATTGTGGAAGG-3‘	68 bp
Gapdh, rev	5‘- ATCCACAGTCTTCTGGGTGG-3‘	
