# Supplementary material for: Regulation of Microtubule Nucleation in Mouse Bone Marrow-Derived Mast Cells by Protein Tyrosine Phosphatase SHP-1
Source: Cells. 2019 Apr 11;8(4):345. doi: 10.3390/cells8040345 (PMC6523986; doi:10.3390/cells8040345)
Supplement: Supplementary file 1 [file cells-08-00345-s001.zip › Text S1.pdf]

Supplementary Text S1

## Features of in-house written macro for processing images from microtubule regrowth experiment

Images of γ-tubulin (centrosomal marker, 1st channel) and α-tubulin (microtubule asters, 2nd channel) were stored separately as z-stacks in tif format. The size of the image matrix was 256 × 256 pixels, using 16-bit depth. The size of voxel was 0.106 µm × 0.106 µm × 200 µm. Macro written in ImageJ macro language using Fiji works under a directory with files in tif format to process automatically all contained images.

Tasks for individual channels

1st channel: Creating Maximum Intensity projection from a z-stack data. Smoothing the resulting picture by a 5x5 mean filter to remove high-intensity noise particles. Finding the brightest point in the smoothed picture corresponding to a center of a centrosome. Creating a circle 1 µm in a diameter as a ROI around this center. Measuring a mean intensity in this circle in original non-smoothed data.

2nd channel: Creating Maximum Intensity projection from a z-stack data. Removing non-homogenous background from this picture. Creating a circle 1 µm in a diameter as a ROI around the center point position found in the 1st channel. Measuring a mean intensity in this circle.

For checking the quality of analysis after the computation, an image pair of both channels is stored with overlays of found ROIs for each original picture. Computed mean image intensities are stored in an Excel file.
